# Pharmacologic inhibition of serotonin htr2b ameliorates hyperglycemia and the altered expression of hepatic FGF21, Sdf2l1, and htr2a in db/db mice and KKA^y^ mice

**DOI:** 10.1016/j.heliyon.2020.e05774

**Published:** 2020-12-19

**Authors:** Katsunori Nonogaki, Takao Kaji

**Affiliations:** Laboratory of Diabetes and Nutrition, Tohoku University New Industry Creation Hatcher Center, Japan

**Keywords:** htr2b, htr2a, FGF21, Sdf2l1, Insulin, db/db mice, KKA^y^ mice, Biological sciences, Endocrinology, Pathophysiology, Health sciences, Physiology, Pharmacology, Internal medicine

## Abstract

Plasma fibroblast growth factor 21 (FGF21) levels and hepatic FGF21, serotonin 2a receptor (htr2a), and stromal cell-derived factor 2 like 1 (Sdf2l1) expression are increased in insulin-resistant C57BL6J mice fed a high-fat diet. Here we show that plasma FGF21 levels and hepatic FGF21, Sdf2l1, and htr2a expression were decreased in 6-week-old db/db mice compared with C57BL6J mice, whereas they were increased in 6-week-old KKA^y^ mice compared with KK mice. Expression of hepatic htr2b was increased in db/db mice and KKA^y^ mice compared with controls. Treatment with the selective htr2b antagonist SB204741 suppressed the hyperglycemia in either db/db mice or KKA^y^ mice. Treatment with SB20471 reversed the decreases in plasma FGF21 levels and hepatic FGF21, Sdf2l1, and htr2a expression in db/db mice, whereas it suppressed the increases in plasma FGF21 levels and hepatic FGF21, Sdf2l1, and htr2a expression in KKA^y^ mice. Moreover, treatment with SB204741 increased plasma FGF21 levels and expression of hepatic FGF21, htr2a, and Sdf2l1 in C57BL6J mice, whereas it decreased plasma FGF21 levels and hepatic FGF21 expression in KK mice. These findings suggest that pharmacologic inhibition of htr2b ameliorates the hyperglycemia and altered expression of hepatic FGF21, Sdf2l1 and htr2a in obese and diabetic db/db and KKA^y^ mice.

## 1 Introduction

Serotonin (5-HT) is not only a neurotransmitter but also an endocrine hormone secreted by the gut and peripheral organs that promotes the efficient storage of energy by upregulating lipid anabolism [1]. Recent studies demonstrated that pharmacologic and genetic inhibition of peripheral 5-HT synthesis suppresses high-fat diet-induced obesity, hepatic steatosis and glucose intolerance in mice [2, 3, 4].

The 5-hydeoxytryptamine 2b receptor (htr2b) is highly expressed in the liver and its expression increases upon fasting [4]. Gut-derived 5-HT promotes hepatic gluconeogenesis and inhibits glucose uptake through htr2b in hepatocytes [4]. A recent study, however, demonstrated that hepatic expression of the 5-HT2a receptor (htr2a), but not htr2b, is increased in obese mice fed a high-fat diet for 8 weeks [5], and either genetic ablation of liver-specific htr2a or treatment with a selective ht2a antagonist suppresses hepatic steatosis in mice fed a high-fat diet for 8 weeks. Htr2a in the liver may therefore contribute to hepatic steatosis in mice fed a high-fat diet [5].

FGF21 is primarily secreted by the liver as an endocrine hormone [6]. Although FGF21 has several beneficial effects on insulin sensitivity and glucose and lipid metabolism [6, 7], circulating FGF21 levels are paradoxically increased in hepatosteatosis, obesity, and type 2 diabetes in rodents and humans [6, 7, 8, 9, 10, 11, 12, 13]. Our previous study demonstrated that plasma FGF21 levels and hepatic FGF21 expression are decreased in old obese and diabetic db/db mice with leptin receptor mutation compared with age-matched C57BL6J mice [14], although these are increased in obese C57BL6J mice fed a high-fat diet [15].

Treatment with a tryptophan hydroxylase inhibitor, which inhibits 5-HT synthesis, reversed the alterations of hepatic FGF21 production in obese mice fed a high-fat diet and old db/db mice, suggesting that 5-HT may contribute to the regulation of hepatic FGF21 production in obese and diabetic mice [14, 15]. Moreover, our recent studies demonstrated that plasma FGF21 levels and hepatic FGF21 expression are decreased in tryptophan hydroxylase 1 (Tph1)-deficient mice, which display a remarkable low levels of plasma 5-HT [16]. Thus, peripheral 5-HT is essential to maintain plasma FGF21 levels and hepatic FGF21 expression [16].

Normal glucose and lipid homeostasis require endoplasmic reticulum (ER) stress responses in the liver following feeding that are terminated by stromal cell-derived factor 2 like 1 (Sdf2l1) [17]. Expression of hepatic Sdf2l1 is decreased in fasted state and is increased by refeeding [17]. Moreover, expression of hepatic Sdf2l1 is decreased in obese and diabetic db/db mice and humans, and the suppression of hepatic Sdf2l1 expression results in insulin resistance and hepatic steatosis with a sustained ER stress [17]. Thus, alterations of hepatic FGF21 and Sdf2l1 expression may be parallel in obese and diabetic db/db mice.

On the other hand, our recent studies demonstrated that expression of hepatic FGF21 and Sdf2l1 is increased in insulin-resistant C57BL6J mice fed a high-fat diet for 13 days, and that the suppression of hepatic FGF21 and Sdf2l1 expression induced by whey protein insolate decreases insulin resistance and hyperglycemia in C57BL6J mice fed a high-fat diet [16]. Alterations of hepatic htr2a and htr2b expression in obese and diabetic mice and role of htr2b in the regulation of hepatic htr2a, FGF21 and Sdf2l1 expression and glucose metabolism, however, have not been evaluated.

SB204741 is a highly specific molecular antagonist of htr2b that has negligible effects on htr2a or htr2c [18]. Intraperitoneal injection of SB204741 reportedly exerts an antifibrogenic effect via htr2b in a model of progressive liver fibrosis [19]. In the present study, we examined plasma FGF21 levels and the expression of hepatic FGF21, Sdf2l1, htr2a, and htr2b in young db/db mice with leptin receptor mutation and KKA^y^ with ectopic expression of agouti peptide, compared with age-matched control mice. In addition, we examined the effect of intraperitoneal injection of the selective htr2b antagonist SB204741 on blood glucose levels, plasma FGF21 and insulin levels, and expression of hepatic FGF21, Sdf2l1, and htr2a in obese and diabetic db/db mice and KKA^y^ mice in vivo.

## 2 Materials and methods

### 2.1 General procedures

Male C57BL6J mice, db/db mice, KK and KKA^y^ mice (5 weeks old) were purchased from Japan CLEA. The mice were individually housed in cages with free access to water and chow pellets in a light- and temperature-controlled environment (12 h on/12 h off, lights on at 08:00; 20–22 °C).

In the first experiment, 6-week-old db/db mice and C57BL6J mice were decapitated, and blood was obtained for the measurement of plasma FGF21 and insulin levels. The liver was dissected for determining mRNA levels.

In the second experiment, 6-week-old KKA^y^ mice and KK mice were decapitated, and blood was obtained for the measurement of plasma FGF21 and insulin levels. The liver was dissected for determining mRNA levels.

Finally, these animals were intraperitoneally injected with vehicle or SB204741 (5 mg/kg), a selective htr2b antagonist, once daily for 3 days. Daily food intake and body weight changes were determined. At the fourth day, the animals were decapitated and blood was obtained for the measurement of blood glucose, plasma insulin and FGF21 levels. The liver was excluded for determining mRNA levels.

The experiments were performed between 14:00–16:00. The SB202741 was dissolved in 0.2 ml 5% DMSO saline (vehicle). The dose of SB202741 (5 mg/kg) was selected based on evidence that SB202741 attenuated fibrogenesis and improved liver function in disease models in which fibrosis was pre-established and progressive in vivo [19].

The animal studies were conducted in accordance with the institutional guidelines for animal experiments at Tohoku University Graduate School of Medicine. All experimental protocols and animal ethics were approved by the institutional committee at Tohoku University.

### 2.2 Blood chemistry

Whole blood was mixed with EDTA-2Na (2 mg/ml) and aprotinin (500 kIU/ml) to determine the plasma levels of FGF21. Plasma levels of FGF21 and insulin were measured by enzyme-linked immunosorbent assay (rat/mouse FGF21 ELISA Kit, R&D Systems, Tokyo, Japan; and a mouse Insulin ELISA Kit [TMB], AKRIN-011T, Shibayagi, Gunma, Japan, respectively) as described previously [16]. Blood glucose levels were measured using glucose strips (blood glucose monitoring system; Accu-Check, Roche Diagnostics, Tokyo, Japan).

### 2.3 Real-time quantitative reverse transcription–polymerase chain reaction (RT–PCR)

Total RNA was isolated from mouse liver using the RNeasy Midi kit (Qiagen, Hilden, Germany) according to the manufacturer's instructions. cDNA synthesis was performed using a Super Script III First-Strand Synthesis System for RT-PCR Kit (Invitrogen, Rockville, MD) with 1 μg total RNA. cDNA synthesized from total RNA was evaluated in a real-time PCR quantitative system (LightCycler Nano Instrument Roche Diagnostics, Mannheim, Germany). The primers were listed in Table 1.

**Table 1:** Primers used in qRT-PCR analysis.

GENE		SEQUENCE
FGF21	Sense	CACCGCAGTCCAGAAAGTC
	antisense	ATCAAAGTGAGGCGATCCA
Sdf2l1	Sense	CACACGGTCCAATAGCAGTG
	antisense	GCTCTAGACCTCTGCGCTTC
Htr2a	sense	TTCAGTGCCAGTACAAGGAG
	antisense	GAGTGTTGGTTCCCTAGTGTAA
Htr2b	sense	CAATCATCCTCCTCGATACCC
	antisense	GAAGCCATCAGATCTACTTTAGCC
β-actin	sense	TTGTAACCAACTGGGACGATATGG
	antisense	GATCTTGATCTTCATGGTGCTAGG

The relative amount of mRNA was calculated using β-actin mRNA as the invariant control. Data are shown as fold-change of the mean value of the control group, which received saline as described previously [16, 20, 21].

## 3 Statistical methods

Data are presented as mean ± SEM (n = 6). Comparisons between two groups were performed using Student's t-test. Comparisons between more than two groups were performed using analysis of variance with Bonferroni's correction for multiple comparisons. A P value of less than 0.05 was considered statistically significant.

## 4 Results

### 4.1 Plasma FGF21 levels and altered expression of hepatic FGF21, Sdf2l1, htr2a, and htr2b in db/db mice

**Figure 1:**
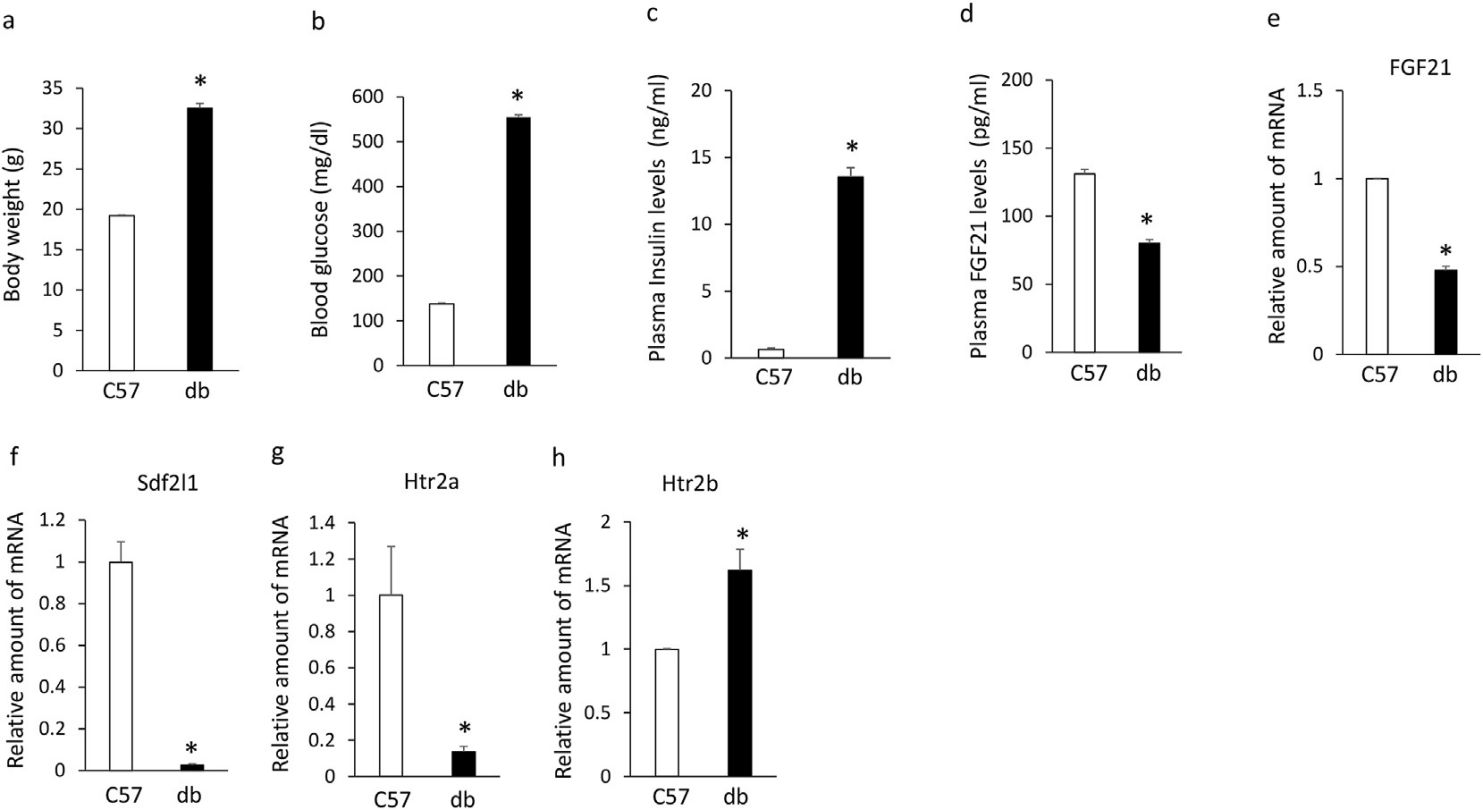
Body weight (a), blood glucose levels (b), plasma insulin (c) and FGF21 (d) levels, expression of hepatic FGF21 (e), Sdf2l1 (f), htr2a (g), and htr2b (h) in 6-week-old db/db mice compared with C57BL6J mice. Data are presented as the mean ± SEM (n = 6/group). ∗P < 0.05.

Although body weights (Figure 1a), blood glucose levels (Figure 1b) and plasma insulin levels (Figure 1c) were significantly increased in db/db mice compared with C57BL6J mice, plasma FGF21 levels (Figure 1d) and expression of hepatic FGF21 (Figure 1e) were significantly decreased in db/db mice compared with C57BL6J mice. In addition, expression of hepatic Sdf2l1 (Figure 1f) and htr2a (Figure 1g) was remarkably decreased in db/db mice compared with C57BL6J mice, whereas expression of hepatic htr2b was significantly increased in db/db mice compared with C57BL6J mice (Figure 1 h).

### 4.2 Effects of SB204741 on plasma FGF21 levels and the expression of hepatic FGF21, Sdf2l1, htr2a, and htr2b in db/db mice

**Figure 2:**
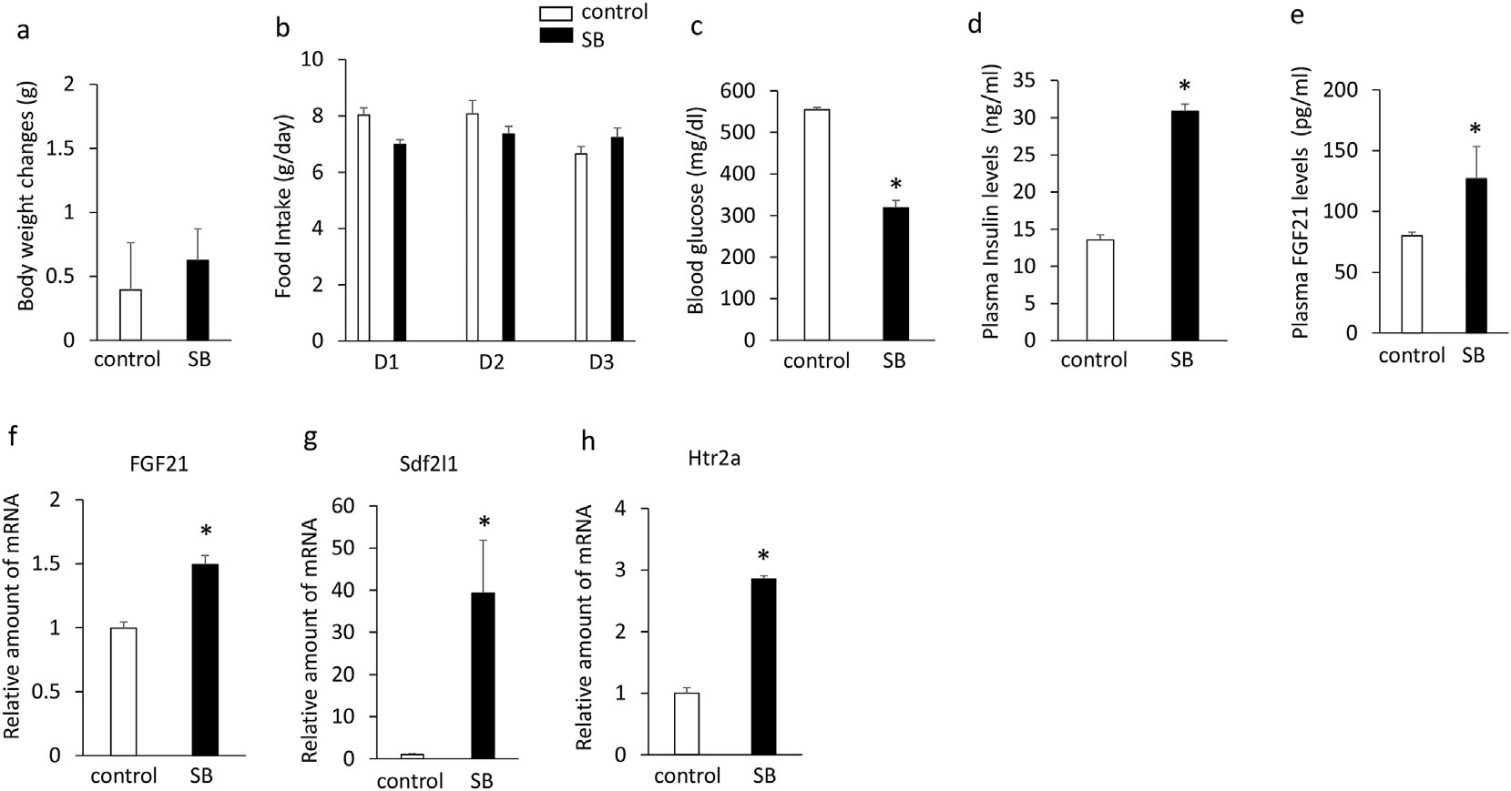
Effects of intraperitoneally injection of SB204741 (5 mg/kg) or vehicle once daily for 3 days on body weight changes (a), daily food intake (b), blood glucose levels (c), plasma insulin (d) and FGF21 (e) levels, expression of hepatic FGF21 (f), Sdf2l1 (g), and htr2a (h) in 6-week-old db/db mice. Basal body weights in db/db mice were 32.8g ± 0.7g (controls) and 31.9 ± 0.2 g (SB204741 group), respectively. Data are presented as the mean ± SEM (n = 6/group). ∗P < 0.05. C; controls, SB; SB204741.

Although treatment with SB204741 for 3 days had no significant effects on body weight (Figure 2a) and daily food intake (Figure 2b), it significantly decreased blood glucose levels in db/db mice (Figure 2c). In addition, treatment with SB204741 significantly increased plasma insulin (Figure 2d) and FGF21 (Figure 2e) levels and expression of hepatic FGF21 (Figure 2f), Sdf2l1 (Figure 2g), and htr2a (Figure 2h) compared with controls. These findings suggest that treatment with the selective htr2b antagonist enhances hyperinsulinemia and suppresses hyperglycemia and the decreases in plasma FGF21 levels and hepatic FGF21, Sdf2l1, and htr2a expression in db/db mice.

### 4.3 Effects of SB204741 on plasma FGF21 levels and the expression of hepatic FGF21, Sdf2l1, htr2a, and htr2b in C57BL6J mice

**Figure 3:**
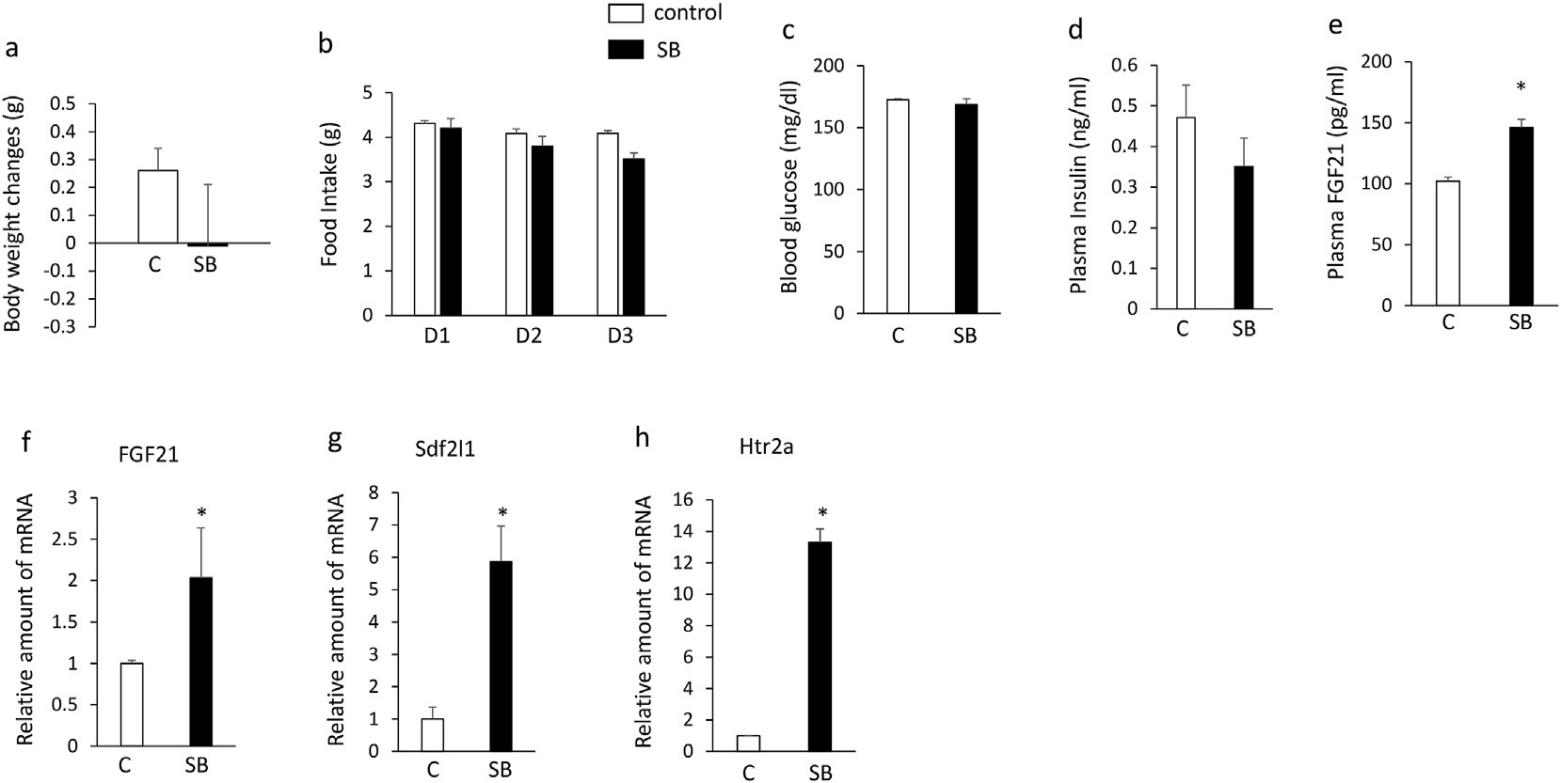
Effects of intraperitoneally injection of SB204741 (5 mg/kg) or vehicle once daily for 3 days on body weight changes (a), daily food intake (b), blood glucose levels (c), plasma insulin (d) and FGF21 (e) levels, expression of hepatic FGF21 (f), Sdf2l1 (g), and htr2a (h) in 6-week-old C57BL6J mice. Basal body weights in C57BL6J mice were 20.5g ± 0.2g (controls) and 20.0 ± 0.6 g (SB204741 group), respectively. Data are presented as the mean ± SEM (n = 6/group). ∗P < 0.05. C; controls, SB; SB204741.

Although treatment with SB204741 for 3 days had no significant effects on body weight (Figure 3a), daily food intake (Figure 3b), blood glucose levels (Figure 3c) and plasma insulin levels (Figure 3d), it significantly increased plasma FGF21 levels (Figure 3e) and expression of hepatic FGF21 (Figure 3f), Sdf2l1 (Figure 3g) and htr2a (Figure 3h) compared with controls in C57BL6J mice. These findings suggest that treatment with the selective htr2b antagonist increases plasma FGF21 levels and expression of hepatic FGF21, Sdf2l1, and htr2a in C57BL6J mice.

### 4.4 Plasma FGF21 levels and altered expression of hepatic FGF21, Sdf2l1, htr2a, and htr2b in KKA^y^ mice

**Figure 4:**
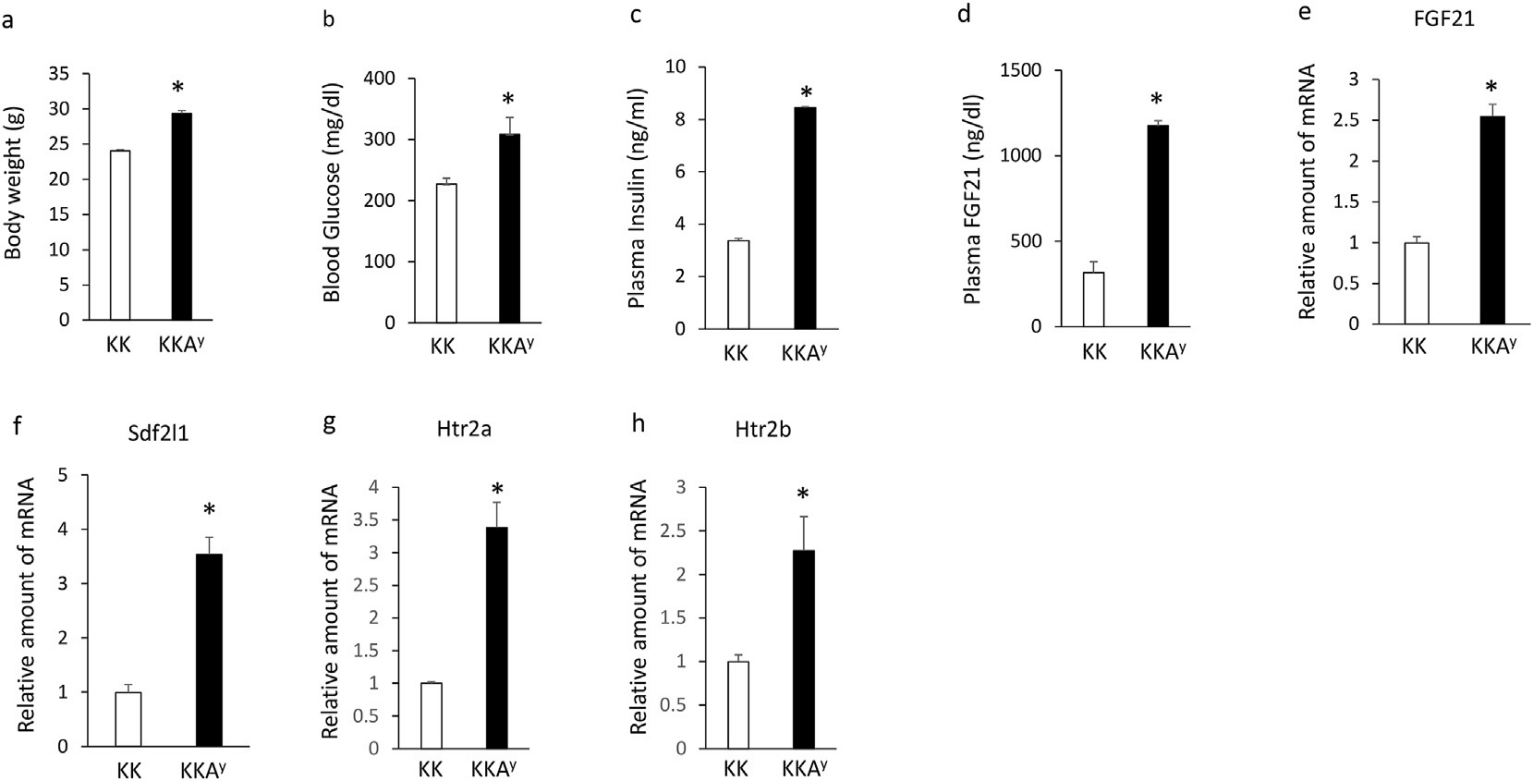
Body weight (a), blood glucose levels (b), plasma insulin (c) and FGF21 (d) levels, expression of hepatic FGF21 (e), Sdf2l1 (f), htr2a (g) and htr2b (h) in 6-week-old KKA^y^ mice compared with KK mice. Data are presented as the mean ± SEM (n = 6/group). ∗P < 0.05.

Although body weights (Figure 4a), blood glucose levels (Figure 4b), plasma insulin (Figure 4c) and FGF21 (Figure 4d) levels and expression of hepatic FGF21 (Figure 4e), Sdf2l1 (Figure 4f), htr2a (Figure 4g) and htr2b (Figure 4h) were significantly increased in KKA^y^ mice compared with KK mice. These findings suggest that agouti peptide upregulates plasma FGF21 levels and expression of hepatic FGF21, Sdf2l1, htr2a and htr2b in KK mice.

### 4.5 Effects of SB204741 on plasma FGF21 levels and the expression of hepatic FGF21, Sdf2l1, htr2a, and htr2b in KKA^y^ mice

**Figure 5:**
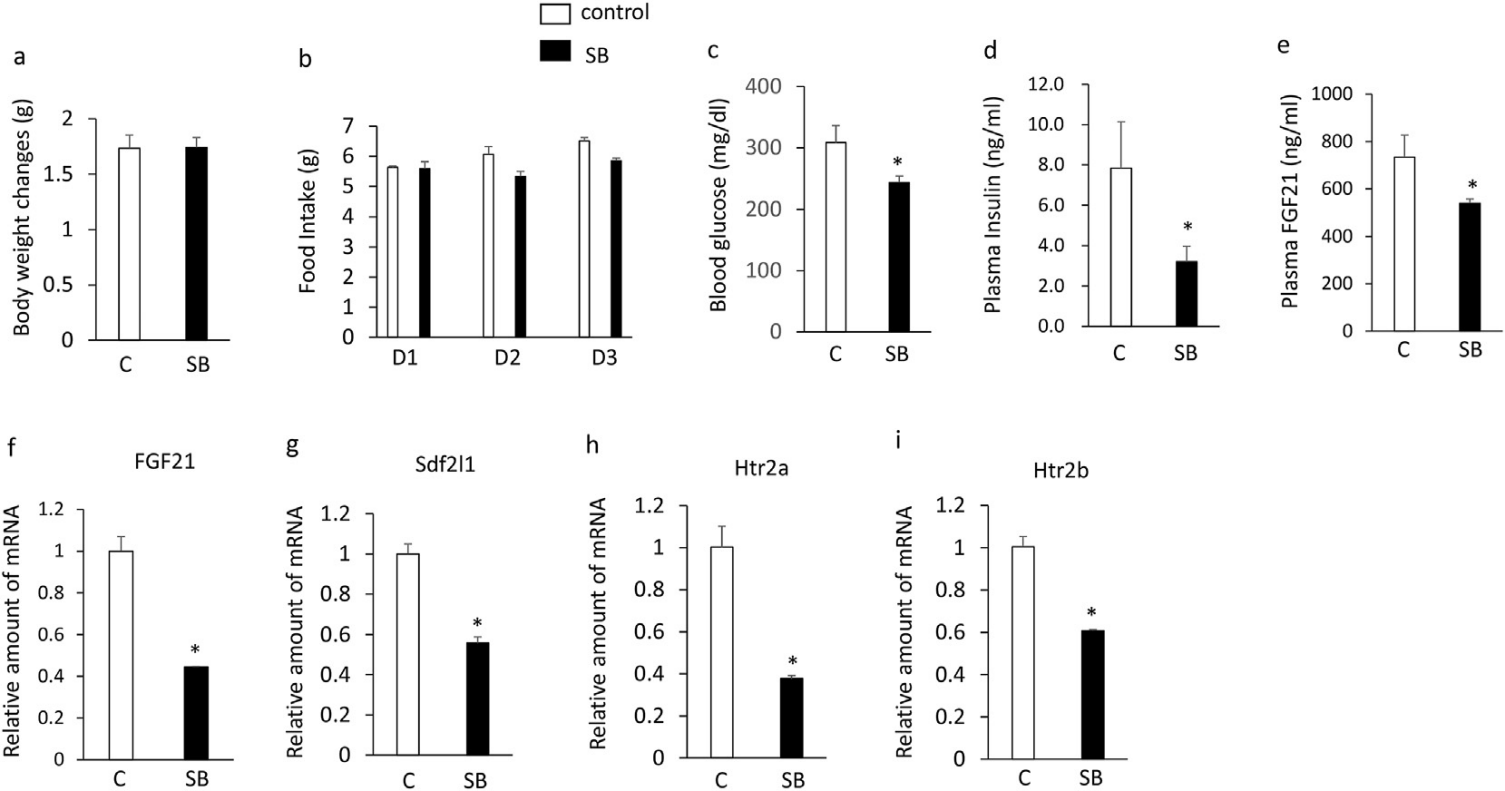
Effects of intraperitoneally injection of SB204741 (5 mg/kg) or vehicle once daily for 3 days on body weight changes (a), daily food intake (b), blood glucose levels (c), plasma insulin (d) and FGF21 (e) levels, expression of hepatic FGF21 (f), Sdf2l1 (g), htr2a (h), (i) and htr2b (i) in 6-week-old KKA^y^ mice. Basal body weights in KKA^y^ mice for 3 days were 26.9g ± 0.3g (controls) and 27.0 ± 0.5 g (SB204741 group), respectively. Data are presented as the mean ± SEM (n = 6/group). ∗P < 0.05. C; controls, SB; SB204741.

Although treatment with SB204741 for 3 days had no significant effects on body weight (Figure 5a) and daily food intake (Figure 5b), it significantly suppressed blood glucose levels (Figure 5c), plasma insulin (Figure 2d) and FGF21 levels (Figure 5e), and overexpression of hepatic FGF21 (Figure 5f), Sdf2l1 (Figure 5g), htr2a (Figure 5h) and htr2b (Figure 5i) compared with controls in KKA^y^ mice. These findings suggest that treatment with the selective htr2b antagonist suppresses hyperglycemia, hyperinsulinemia, the increases in plasma FGF21 levels and hepatic FGF21, Sdf2l1, and htr2a expression in KKA^y^ mice.

### 4.6 Effects of SB204741 on plasma FGF21 levels and the expression of hepatic FGF21, Sdf2l1, htr2a, and htr2b in KK mice

**Figure 6:**
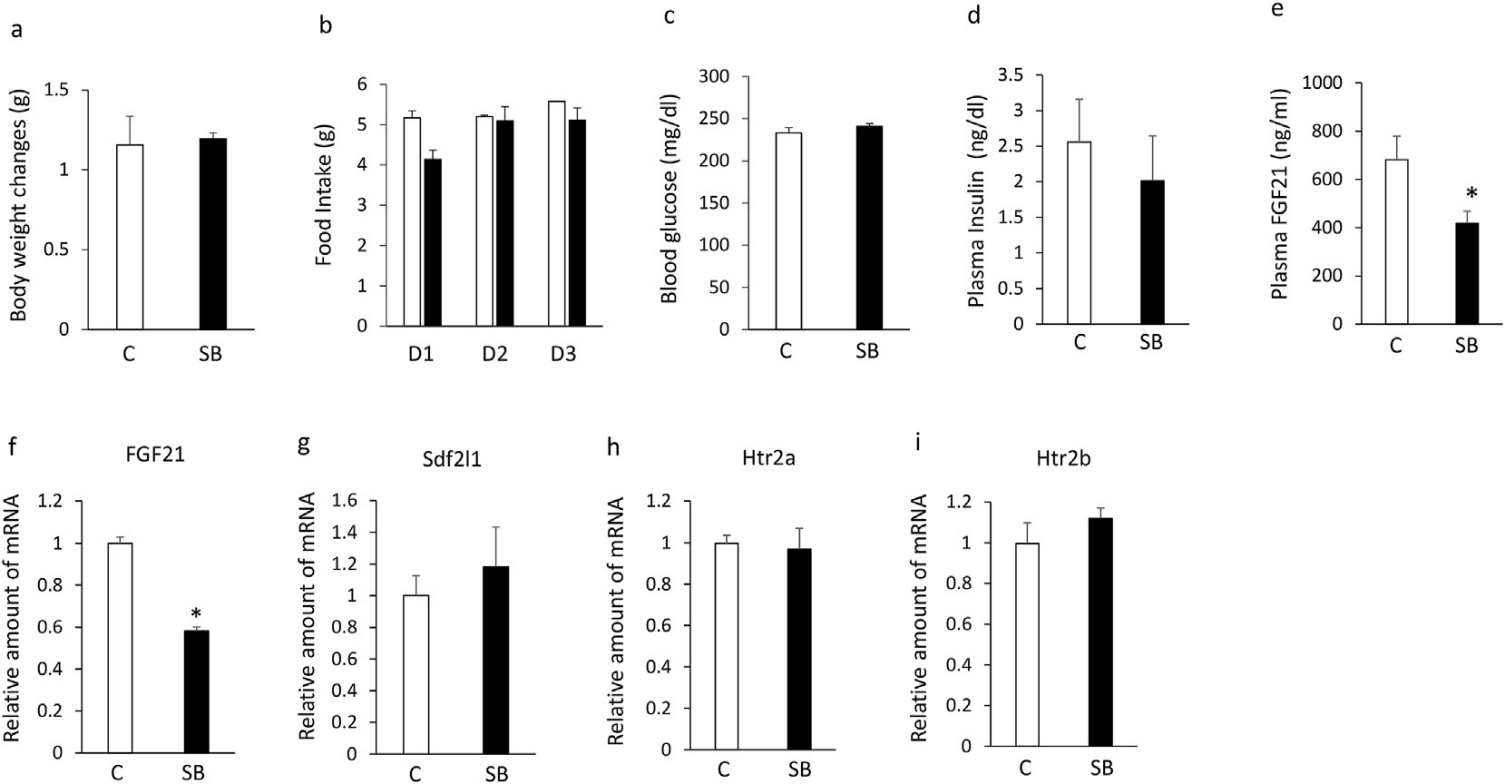
Effects of intraperitoneally injection of SB204741 (5 mg/kg) or vehicle once daily for 3 days on body weight changes (a), daily food intake (b), blood glucose levels (c), plasma insulin (d) and FGF21 (e) levels, expression of hepatic FGF21 (f), Sdf2l1 (g), htr2a (h), and htr2b (i) in 6-week-old KK mice. Basal body weights in KK mice were 25.3g ± 0.2g (controls) and 25.2 ± 0.2 g (SB204741 group), respectively. Data are presented as the mean ± SEM (n = 6/group). ∗P < 0.05. C; controls, SB; SB204741.

Although treatment with SB204741 for 3 days had no significant effects on body weight (Figure 6a), daily food intake (Figure 6b), blood glucose levels (Figure 6c), and plasma insulin levels (Figure 6d), it significantly decreased plasma FGF21 levels (Figure 6e) and expression of hepatic FGF21 (Figure 6f) compared with controls in KK mice. The treatment with SB204741 had no significant effects on expression of hepatic Sdf2l1 (Figure 6g), htr2a (Figure 6h) and htr2b (Figure 6i). These findings suggest that treatment with the selective htr2b antagonist decreases plasma FGF21 levels and expression of hepatic FGF21 in KK mice.

## 5 Discussion

Lethal yellow (A^y^) mice have dominant alleles at the agouti locus (A), which produces ectopic expression of the agouti peptide, an antagonist of the hypothalamic melanocortin (MC)-4 receptors and MC-3 receptors, and display obesity and pronounced diabetes, when bred with KK mice [22, 23, 24]. Our present results revealed that plasma FGF21 levels and hepatic FGF21 expression were increased in KKA^y^ mice compared with KK mice. We recently reported that plasma FGF21 levels and hepatic FGF21 expression are increased in insulin-resistant C57BL6J mice fed a high-fat diet compared with a chow diet for 13 days [16]. Thus, the agouti peptide and a high-fat diet might have similar effects on plasma FGF21 levels and hepatic FGF21 expression.

On the other hand, our previous [14] and present results demonstrated that despite obesity and hyperglycemia, plasma FGF21 levels were decreased in young (6-week-old) and old (9-week-old) db/db mice compared with C57BL6J mice fed a chow diet. Plasma FGF21 levels were lower in db/db mice than in C57BL6J mice fed a high-fat diet [16], KKA^y^ and KK mice. Thus, impaired leptin receptor signaling and a high-fat diet or agouti peptide might have different effects on plasma FGF21 levels and hepatic FGF21 expression.

So et al. [25] reported that serum FGF21 levels are increased in db/db mice compared with controls. The discrepancy between the report by So et al. [25] and our results are likely due to differences in the controls. Moreover, the difference might be due to the age of the mice. In So et al. [25], the serum FGF21 levels were increased in 12-week-old db/db mice compared with heterozygous db/m mice, whereas there were no significant differences in the serum FGF21 levels between 4-week-old or 8-week-old db/db mice and heterozygous db/m mice. Because heterozygous db/m mice, which have low serum FGF21 levels, were used as controls in their report [25], the serum FGF21 levels were increased in 12-week-old db/db mice. In any event, both the previous results [25] and our results demonstrate that the circulating FGF21 levels in db/db mice are actually low.

In addition, our results demonstrated that expression of hepatic Sdf2l1 and htr2a was decreased in db/db mice compared with C57BL6J mice, whereas it was increased in KKA^y^ mice or C57BL6J mice fed a high-fat diet [16] compared with controls. Thus, the alterations of hepatic FGF21, Sd2l1, and htr2a expression were parallel in these diabetic mice. Pharmacologic stimulation of htr2a expression induces the expression of Sdf2l1 and FGF21 in C57BL6J mice [16]. The opposite alterations of hepatic htr2a expression might therefore induce the opposite alterations of hepatic Sdf2l1 and FGF21 in db/db mice and KKA^y^ mice.

In contrast to hepatic htr2a expression, our results demonstrated that the expression of hepatic htr2b was increased in KKA^y^ mice and db/db mice compared with the controls. Moreover, treatment with the selective htr2b antagonist SB204741 suppressed hyperglycemia in db/db mice and KKA^y^ mice without reducing body weight. The effects of the selective htr2b antagonist on plasma FGF21 levels and the expression of hepatic FGF21, Sdf2l1, and htr2a, however, were opposite between db/db mice and KKA^y^ mice. The opposite effects of the selective htr2b antagonist on the expression of hepatic FGF21, Sdf2l1, and htr2a might be due to differences in mice with the C57BL and KK backgrounds. Thus, the mouse strain could alter the effects of the selective htr2b antagonist on plasma FGF21 levels and the expression of hepatic FGF21, Sdf2l1, and htr2a.

A report by Sasako et al. [17] suggested that insufficient expression of Sdf2l1 results in insulin resistance in obese and diabetic db/db mice. Restoration of Sdf2l1 expression by administration of Ad-Sdf2l1 ameliorates insulin resistance and glucose tolerance in db/db mice [17]. Our results in db/db mice support their finding of insufficient expression of Sdf2l1 in db/db mice. In addition, our results demonstrated that treatment with a selective htr2b antagonist rescued the insufficient expression of hepatic Sdf2l1 and increased plasma insulin levels in db/db mice. Thus, the effects of a selective htr2b antagonist on plasma insulin levels are different than the restoration of Sdf2l1 expression by administration of Ad-Sdf2l1 in db/db mice.

Overexpression of FGF21 in the β-cells of pancreatic islets leads to increases in insulin secretion and plasma insulin levels in db/db mice, but not in control mice [26]. Increased hepatic FGF21 production by treatment with the selective htr2b antagonist may contribute to the increases in insulin secretion and plasma insulin levels in db/db mice. We also cannot rule out the direct effect of the selective htr2b antagonist on the β-cells of pancreatic islets.

In summary, the present findings suggest that expression of the hepatic htr2b is increased in young diabetic db/db mice and KKA^y^ mice, and pharmacologic inhibition of htr2b ameliorates the hyperglycemia and altered expression of hepatic FGF21, Sdf2l1 and htr2a in these mice.

## Declarations

### Author contribution statement

Katsunori Nonogaki-Conceived and designed the experiments; Performed the experiments; Analyzed and interpreted the data; Contributed reagents, materials, analysis tools or data; Wrote the paper.

Takao Kaji-Performed the experiments; Analyzed and interpreted the data.

### Funding statement

This research did not receive any specific grant from funding agencies in the public, commercial, or not-for-profit sectors.

### Data availability statement

Data included in article referenced in article.

### Declaration of interests statement

The authors declare no conflict of interest.

### Additional information

No additional information is available for this paper.

## References

[1] Yabut J.M., Crane J.D., Green A.E., Keating D.J., Khan W.I., Steinberg G.R. (2019). Emerging roles for serotonin in regulating metabolism: new implications for an ancient molecule. Endocr. Rev..

[2] Crane J.D., Palanivel R., Mottillo E.P., Bujak A.L., Wang H., Ford R.J., Collins A., Blumer R.M., Fullerton M.D., Yabut J.M., Kim J.J., Ghia J.E., Hamza S.M., Morrison K.M., Schertzer J.D., Dyck J.R.B., Khan W.I., Steinberg G.R. (2015). Inhibiting peripheral serotonin synthesis reduces obesity and metabolic dysfunction by promoting brown adipose tissue thermogenesis. Nat. Med..

[3] Oh C.M., Namkung J., Go Y., Shong K.E., Kim K., Kim H., Park B.Y., Lee H.W., Jeon Y.H., Song J., Shong M., Yadav V.K., Karsenty G., Kajimura S., Lee I.K., Park S., Kim H. (2015). Regulation of systemic energy homeostasis by serotonin in adipose tissues. Nat. Commun..

[4] Sumara G., Sumara O., Kim J.K., Karsenty G. (2012). Gut-derived serotonin is a multifunctional determinant to fasting adaptation. Cell Metabol..

[5] Choi W., Namkung J., Hwang I., Kim H., Lim A., Park H.J., Lee H.W., Han K.H., Park S., Jeong J.S., Bang G., Kim Y.H., Yadav V.K., Karsenty G., Ju Y.S., Choi C., Suh J.M., Park J.Y., Park S., Kim H. (2018). Serotonin signals through a gut-liver axis to regulate hepatic steatosis. Nat. Commun..

[6] Markan K.R., Naber M.C., Ameka M.K., Anderegg M.D., Mangelsdorf D.J., Kliewer S.A., Mohammadi M., Potthoff M.J. (2014). Circulating FGF21 is liver derived and enhances glucose uptake during refeeding and overfeeding. Diabete.

[7] Kharitonenkov A., DiMarchi R. (2015). FGF21 revolutions: recent advances illuminating FGF21 biology and medicinal properties. Trends Endocrinol. Metabol..

[8] Zhang X., Yeung D.C.Y., Karpisek M., Stejskal D., Zhou Z.G., Liu F., Wong R.L.C., Chow W.S., Tso A.W.K., Lam K.S.L., Xu A. (2008). Serum FGF21 levels are increased in obesity and are independently associated with the metabolic syndrome in humans. Diabete.

[9] Charvez A.O., Molina-Carrion M., Abdul-Ghani M.A., Folli F., Defronzo R.A., Tripathy D. (2009). Circulating fibroblast growth factor-21 is elevated in impaired glucose tolerance and type 2 diabetes and correlates with muscle and hepatic insulin resistance. Diabet. Care.

[10] Mraz M., Bartlova M., Lacinova Z., Michalsky D., Kasalicky M., Haluzikova D., Matoulek M., Dostalova I., Humenanska V., Haluzik M. (2009). Serum concentrations and tissue expression of a novel endocrine regulator fibroblast growth factor-21 in patients with type 2 diabetes and obesity. Clin. Endocrinol..

[11] Dushay J., Chui P.C., Gopalakrishnan G.S., Varela-Rey M., Crawley M., Fisher F.M., Badman M.K., Martinez-Chantar M.L., Maratos-Flier E. (2010). Increased fibroblast growth factor 21 in obesity and nonalcoholic fatty liver disease. Gastroenterol..

[12] Fisher F.M., Chui P.C., Antonellis P.J., Bina H.A., Kharitonenkov A., Flier J.S., Maratos-Flier E. (2010). Obesity is a fibroblast growth factor 21(FGF21)-resistant state. Diabete.

[13] Chen C., Cheung B.M.Y., Tso A.W.K., Wang Y., Law L.S.C., Ong K.L., Wat N.M.S., Xu A., Lam K.S.L. (2011). High plasma level of fibroblast growth factor 21 is an independent predictor of type 2 diabetes. Diabet. Care.

[14] Nonogaki K., Kaji T., Murakami M. (2018). A tryptophan hydroxylase inhibitor increases hepatic FGF21 production and decreases hepatic gluconeogenesis independently of insulin in db/db mice. Neuropsychiatry.

[15] Nonogaki K., Kaji T., Murakami M. (2018). A tryptophan hydroxylase inhibitor decreases hepatic FGF21 expression and circulating FGF21 in mice fed a high-fat diet. Neuropsychiatry.

[16] Nonogaki K., Kaji T. (2020). Whey protein isolate inhibits hepatic FGF21 production, which precedes weight gain, hyperinsulinemia and hyperglycemia in mice fed a high-fat diet. Sci. Rep..

[17] Sasako T., Ohsugi M., Kubota N., Itoh S., Okazaki Y., Terai A., Kubota T., Yamashita S., Nakatsukasa K., Kamura T., Iwayama K., Tokuyama K., Kiyonari H., Furuta Y., Shibahara J., Fukayama M., Enooku K., Okushin K., Tsutsumi T., Tateishi R., Tobe K., Asahara H., Koike K., Kadowaki T., Ueki K. (2019). Hepatic Sdf2l1 controls feeding-induced ER stress and regulates metabolism. Nat. Commun..

[18] Forbes I.T., Jones G.E., Murphy O.E., Holland V., Baxter G.S. (1995). N-(1-methyl-5-indolyl)-N′-(3-methyl-5-isothiazolyl)urea: a novel, high-affinity 5-HT2B receptor antagonist. J. Med. Chem..

[19] Ebrahimkhani M.R., Oakley F., Murphy L.B., Mann J., Moles A., Perugorria M.J., Ellis E., Lakey A.F., Burt A.D., Douglass A., Wright M.C., White S.A., Jaffré F., Maroteaux L., Mann D.A. (2011). Stimulating healthy tissue regeneration by targeting the 5-HT₂B receptor in chronic liver disease. Nat. Med..

[20] Nonogaki K., Kaji T., Yamzaki T., Murakami M. (2016). Pharmacologic stimulation of central GLP-1 receptors has opposite effects on the alterations of plasma FGF21 levels induced by feeding and fasting. Neurosci. Lett..

[21] Nonogaki K., Nozue K., Oka Y. (2007). Social isolation affects the development of obesity and type 2 diabetes in mice. Endocrinology.

[22] Fan W., Boston B.A., Kesterson R.A., Hruby V.J., Cone R.D. (1997). Role of melanocortinergic neurons in feeding and the agouti obesity syndrome. Nature.

[23] Ebihara K., Ogawa Y., Katsuura G., Numata Y., Masuzaki H., Satoh N., Tamaki M., Yoshioka T., Hayase M., Matsuoka N., Aizawa-Abe M., Yoshimasa Y., Nakao K. (1998). Involvement of agouti-related protein, an endogenous antagonist of hypothalamic melanocortin receptor, in leptin action. Diabetes.

[24] Nonogaki K., Nozue K., Oka Y. (2006). Hyperphagia alters expression of hypothalamic 5-HT2C and 5-HT1B receptor genes and plasma des-acyl ghrelin levels in A^y^ mice. Endocrinology.

[25] So W.Y., Cheng Q., Chen L., Evans-Molina C., Xu A., Lam K.S.L., Leung P.S. (2013). High glucose represses β-Klotho expression and impairs fibroblast growth factor 21 action in mouse pancreatic islet. Involvement of peroxisome proliferator-activated receptor γ signaling. Diabetes.

[26] Pan Y., Wang B., Zheng J., Xiong R., Fan Z., Ye Y., Zhang S., Li Q., Gong F., Wu C., Lin Z., Li X., Pan X. (2019). Pancreatic fibroblast growth factor 21 protects against type 2 diabetes in mice by promoting insulin expression and secretion in a PI3K/Akt signaling-dependent manner. J. Cell Mol. Med..

